# Fasting Plasma Insulin at 5 Years of Age Predicted Subsequent Weight Increase in Early Childhood over a 5-Year Period—The Da Qing Children Cohort Study

**DOI:** 10.1371/journal.pone.0127389

**Published:** 2015-06-05

**Authors:** Yan Yan Chen, Jin Ping Wang, Ya Yun Jiang, Hui Li, Ying Hua Hu, Kok Onn Lee, Guang Wei Li

**Affiliations:** 1 Endocrinology and Cardiovascular disease Center, Fuwai Hospital, National Center for Cardiovascular Diseases, Chinese Academy of Medical Sciences and Peking Union Medical College, Beijing, China; 2 Cardiology, Da Qing First Hospital, Da Qing, China; 3 Department of Medicine, Yong Loo Lin School of Medicine, National University of Singapore, Singapore, Singapore; 4 Endocrinology, China-Japan Friendship Hospital, Beijing, China; INIA, SPAIN

## Abstract

**Background:**

The association between hyperinsulinemia and obesity is well known. However, it is uncertain especially in childhood obesity, if initial fasting hyperinsulinemia predicts obesity, or obesity leads to hyperinsulinemia through insulin resistance.

**Objective:**

To investigate the predictive effect of fasting plasma insulin on subsequent weight change after a 5-year interval in childhood.

**Methods:**

424 Children from Da Qing city, China, were recruited at 5 years of age and followed up for 5 years. Blood pressure, anthropometric measurements, fasting plasma insulin, glucose and triglycerides were measured at baseline and 5 years later.

**Results:**

Fasting plasma insulin at 5 years of age was significantly correlated with change of weight from 5 to 10 years (ΔWeight). Children in the lowest insulin quartile had ΔWeight of 13.08±0.73 kg compare to 18.39±0.86 in the highest insulin quartile (*P*<0.0001) in boys, and similarly 12.03±0.71 vs 15.80±0.60 kg (*P*<0.0001) in girls. Multivariate analysis showed that the predictive effect of insulin at 5 years of age on subsequent weight gain over 5 years remained statistically significant even after the adjustment for age, sex, birth weight, TV-viewing time and weight (or body mass index) at baseline. By contrast, the initial weight at 5 years of age did not predict subsequent changes in insulin level 5 years later. Children who had both higher fasting insulin and weight at 5 years of age showed much higher levels of systolic blood pressures, fasting plasma glucose, the homeostasis model assessment for insulin resistance (HOMA-IR) and triglycerides at 10 years of age.

**Conclusions:**

Fasting plasma insulin at 5 years of age predicts weight gain and cardiovascular risk factors 5 year later in Chinese children of early childhood, but the absolute weight at 5 years of age did not predict subsequent change in fasting insulin.

## Introduction

Childhood obesity is an important public health issue all over the world, and is of great concern not only for the possible potential effects on adult health and longevity, but also for the potential burden on the global health costs. The prevalence of obese children in Chinese has been increasing over the last 20 years [1, 2]. Childhood obesity can adversely affect many organ systems and often cause serious consequences, including hypertension, dyslipidemia, diabetes, atherosclerosis and increased incidence of coronary heart disease in later life[3–5].

Within this context, childhood insulin resistance and hyperinsulinemia may have important implications on subsequent health outcomes [6, 7]. However, it is uncertain whether obesity leads to hyperinsulinemia or hyperinsulinemia leads to weight gain. In 1962, Neel proposed the “thrifty genotype” hypothesis that hyperinsulinemia increased the efficiency of fat storage and play a causal role in the development of obesity[8–13]. Contrary to the thrifty genotype hypothesis, other researchers have observed that insulin resistance or hyperinsulinemia was associated with lower body weight gain[14], or that hyperinsulinemia was a result of the increased adipose tissue [15, 16]. However, almost all of these studies were carried out in adults with a high prevalence of obesity and type 2 diabetes, and very few studies have investigated the relationship between insulin and weight gain in early childhood. Olalekan E et al reported that fasting hyperinsulinemia caused more weight gain in aged 5–9 to 15–19 in a Pima Indian children study[17]. We are not aware of any previous longitudinal study in Asian children.

The aim of this present study was to examine the effects of fasting insulin on the subsequent change of weight in early childhood, as well as body mass index (BMI) and the percentage of ideal weight for height (WFH) during a 5-year follow-up period. We hypothesized that fasting insulin in early childhood may be a predictive factor of weight gain.

## Materials and Methods

### Subjects

The study was approved by the Bureau of Education of Da Qing city, the participating schools and the Ethics Committee of the China-Japan Friendship Hospital. The same study group performed the original study and the follow-up study 5 years later. The informed consent was obtained verbally from the parents of the study participants before they came for the initial visit (with blood tests on the 5 year old children) and for the subsequent similar follow-up visit 5 years later. The study (including the first visit and the follow-up study) was approved by the City Education Bureau and the Ethics Committee of the China-Japan Friendship Hospital, they also approved the method of verbal consent from parents and guardians in this study. We did not record the verbal consent in the participant documents, but the original consent covered the first visit and the follow-up visit after 5 years later.

In China, the requirement for informed consent for clinical studies from the State Food and Drug Administration (SFDA) started only from 1996, and for the first 4 years, only verbal consent was required. Written consent was implemented as a requirement subsequently in 2000. This study was performed in 1998, and the study group was not required to obtain written documentation of the informed consent by the SFDA. In other Da Qing city papers published in Diabetes Care (1997,20:537–44) and the Lancet (2008,371:1783–9) this was noted also as written documentation of informed consent was also not available since the Da Qing adult study began in 1986.

Four public elementary schools in the Longnan district in Da Qing city, China, took part in this study. All the children enrolling into first grade at 5 years of age were invited to participate in this study. We recruited the entire cohort of 605 children of first grade from four public elementary schools in Da Qing city. The only exclusion criteria were children with type 1 diabetes, thyroid disease and other serious diseases. Of the 605 children recruited for the study in 1998, 424 children (211 boys and 213 girls) were available for follow up 5 years later.

### Anthropometric measurements

All measurements were made by trained nurses when the children were first enrolled at 5 years of age, and the same team made the measurements 5 years later. Participants wore light clothing and took off their shoes. Height was measured to the nearest 0.1 cm by a calibrated wall-mounted stadiometer. Weight was measured to nearest 0.1 kg with an electronic scale. The mean of the three recordings was used. As a BMI chart and z-scores for local children was not available for China (There are no nationwide BMI chart or z-scores for less than 7 years old children in China), the percentage of ideal weight for height (WFH) was obtained for each subject for our analysis using our percentage ideal weight for height chart (Percentage Ideal Weight for height chart, 1993, School Health Service, Ministry of Health, Singapore), which has been used as an acceptable measure of degree of childhood adiposity in previous publish studies[18].

### Blood pressure

After at least 5 minutes of rest, Blood pressure was measured twice using a mercury sphygmomanometer on the right arm of the seated participant, 15 minutes apart. The average of 2 measurements was used in the analyses.

### Laboratory measurements

All blood samples were obtained at baseline (5 years of age) and 5 years later (at 10 years of age). Fasting blood samples were taken and stored on ice until centrifugation. Plasma samples were stored at –70°C for analysis in batches. Fasting plasma glucose (FPG) and triglycerides (TG) were measured in the central lab (Hitachi 7170, Auto-Biochemistry Instruments). Fasting plasma insulin (FI) was measured in the endocrine lab by radioimmunoassay (DSL, USA). All the biochemistry assays were performed in China-Japan Friendship Hospital in Beijing, China. Insulin resistance was calculated using the homeostasis model assessment for insulin resistance (HOMA-IR = Fasting Plasma Glucose (mmol/L) × Fasting Insulin (mIU/L)/22.5).

### Birth weight and TV watching time

Birth weights of the subjects were obtained from birth certificates. Other data were provided by the subject’s accompanying parent who completed a questionnaire at school. Parents provided information on the number of hours per day their child watched TV for each day of the week (to nearest half an hour), and the number of days the child watched TV for that week.

### Statistical Analysis

Statistical Analysis was carried out using SAS software 9.1(SAS Institute, Cary, NC, USA). Mean values and standard errors were calculated for all variables. The percentage of ideal weight for height (WFH) at baseline, the end of 5-year follow-up, and the change in WFH during the 5-year follow-up were obtained from local standard charts for Singapore Chinese children. The children were ranked to quartiles in ascending order based on fasting insulin at 5 years of age (Fins5). General linear model (GLM) of least squares means was used to determine association among variables in boys, girls and all objects. Student’s t-test (with prior testing to ensure that the data had a normal distribution) was used to compare the difference between variables in boys and girls at baseline and the 5-year follow-up. Multivariate regression models were used to determine predictive factors of change in weight and BMI. In the regression model A, gender, birth weight, TV watching time, weight and fasting insulin at baseline were independent variables; in the regression model B, gender, birth weight, TV watching time, BMI and fasting insulin at baseline were included as the independent variables.

## Results

Table 1 shows the characteristics of the boys and girls at entry and 5 years later. Most of the children in this cohort had body weights within the normal range. At baseline, 79.4% of the girls were normal weight (BMI: 13.1–16.5), 13.6% were overweight and 7.0% were underweight; similarly, 72.0% of the boys were normal weight (BMI: 13.4–16.8), 24.2% were overweight and 3.8% were underweight. After 5 years follow-up, 72.9%, 23.5% and 3.6% of the girls, and 66.8%, 29.4% and 3.8% of the boys were normal weight(BMI:13.6–18.8 for girls; BMI: 14.1–20.1 for boys)[19], overweight and underweight, respectively. There were no statistically significant differences in height, weight, BMI, WFH, blood pressure and fasting plasma glucose and fasting insulin between boys and girls at baseline, but TG in girls was higher than in boys(*P* = 0.04). After 5 years, the weight, BMI, and WFH in boys were significant greater than in girls, at the presence of similar height in two gender groups.

**Table 1:** Physical characteristics and biochemical parameters of boys and girls at ages 5 and 10 years.

	5 years old		10 years old	
Variable	Boys	Girls	Boys	Girls
**No.**	211	213	211	213
**Age,year**	5.51±0.04	5.34±0.04	10.51±0.04	10.39±0.04
**Height,cm**	121.32±0.32	119.11±0.34	143.05±0.40	141.83±0.46
**Weight, kg**	23.38±0.28	21.54±0.24	38.17±0.62	34.95±0.52*
**BMI, kg/m2**	15.83±0.14	15.12±0.11	18.52±0.24	17.24±0.19**
**WFH,%**	102.55±0.91	102.34±0.72	104.45±1.32	100.10±1.00**
**SBP, mmHg**	101.63±0.84	100.05±0.84	97.08±0.74	95.95±0.53*
**DBP, mmHg**	66.63±0.72	66.04±0.76	65.63±0.50	63.72±0.51
**FPG,mmol/L**	5.27±0.03	5.13±0.03	5.36±0.03	5.33±0.03
**FI, μU/ml**	4.37±0.22	4.89±0.20	4.50±0.21	4.39±0.23
**TG, mmol/L**	0.52±0.02	0.63±0.03*	0.98±0.03	1.06±0.03*
**HOMA-IR**	1.04±0.06	1.13±0.05	1.08±0.05	1.05±0.06

Abbreviations BMI,body mass index;WFH, percentage of ideal weight for height; SBP, Systolic blood pressure; DBP, Diastolic blood pressure; FPG, fasting plasma glucose; FI, fasting insulin; TG, triglycerides; HOMA-IR, homeostasis model assessment for insulin resistance.

*P<0.05

**P<0.01

We divided the boys and girls into four groups according to fasting insulin quartiles at baseline to analyze the association between fasting insulin and subsequent weight gain during the 5-year follow-up. The weight, BMI and WFH at the entry gradually increased with the increase of fasting insulin in both genders. The children within the highest insulin quartile had significant higher level of weight, BMI and WFH at 5 years of age compare with the lowest insulin quartile group in all subjects and both boys and girls (*P*<0.01). However, there was no statistically significant association between birth weight and the increase of fasting insulin at 5 years of age. Similarly, the weight, BMI and WFH at 10 years of age also increased over the 5 years follow-up with the increase of fasting insulin. When we compared the relationship between fasting insulin at baseline with the change of weight (ΔWeight), BMI (ΔBMI) and WFH (ΔWFH) over the 5-year follow-up, it was found that the change in these variables also significantly increased with the increase of fasting insulin quartiles in all of the children. Compared with the lowest group, the top fasting insulin quartile group had statistically significant higher levels for ΔWeight (*P*<0.0001), ΔBMI (*P*<0.0001), and ΔWFH (*P* = 0.002) (Table 2). A multivariate regression analysis also showed that fasting insulin at 5 years of age were significantly correlated with ΔWeight (*P*<0.0001) after the adjustment of gender, birth weight, TV viewing time and weight at baseline (gender: *P =* 0.65; birth weight: *P =* 0.41; TV viewing time: *P*<0.0001; weight at baseline: *P*<0.0001). When we took the change of BMI as dependent variable, fasting insulin at 5 years of age was still significantly correlated with ΔBMI after adjusting for the same confounders (Table 3).

**Table 2:** Weight, BMI and WFH at 5, 10 years of age and change of them according to fasting insulin quartiles at 5 years of age.

Fasting insulin quartiles at 5 years of age (μU/ml)												
	Boys				Girls				All			
**Variable**	**1.77±0.38**	**2.94±0.42**	**4.83±0.52**	**9.42±3.21**	**1.79±0.39**	**3.05±0.39**	**4.90±0.54**	**8.61±2.37**	**1.77±0.38**	**2.99±0.41**	**4.87±0.53**	**8.95±2.77**
**N**	**62**	**57**	**48**	**45**	**44**	**49**	**59**	**61**	**106**	**106**	**107**	**106**
**Birth weight(kg)**	**3.41±0.06**	**3.42±0.06**	**3.40±0.06**	**3.45±0.07**	**3.33±0.07**	**3.33±0.06**	**3.36±0.06**	**3.31±0.06**	**3.37±0.04**	**3.38±0.04**	**3.38±0.04**	**3.38±0.04**
**Weight5(kg)**	**22.28±0.46**	**22.23±0.48**	**23.13±0.53**	26.64±0.54**	**20.51±0.51**	**20.90±0.49**	**21.32±0.44**	23.03±0.44**	**21.36±0.35**	**21.53±0.34**	**22.24±0.34**	24.72±0.35**
**BMI5(kg/m** ^**2**^ **)**	**15.23±0.25**	**15.34±0.26**	**15.81±0.28**	17.32±0.29**	**14.50±0.24**	**14.94±0.23**	**15.05±0.21**	15.77±0.20**	**14.85±0.17**	**15.12±0.17**	15.44±0.17*	16.49±0.17**
**WFH5 (%)**	**99.09±1.59**	**99.89±1.67**	**102.59±1.81**	110.80±1.87**	**98.44±1.54**	**101.56±1.46**	**101.98±1.33**	106.13±1.31**	**98.73±1.11**	**100.61±1.11**	102.30±1.11*	108.19±1.12**
**Weight10(kg)**	**35.37±1.05**	**35.77±1.09**	**38.28±1.19**	45.03±1.12**	**32.54±1.09**	**33.19±1.04**	**34.19±0.94**	38.83±0.93**	**33.85±0.76**	**34.43±0.75**	36.22±±0.75*	41.75±0.76**
**BMI10(kg/m** ^**2**^ **)**	**17.36±0.42**	**17.60±0.43**	18.74±0.47*	21.08±0.49**	**16.34±0.39**	**16.66±0.37**	**17.03±0.34**	18.55±0.33**	**16.80±0.29**	**17.11±0.29**	17.88±0.29**	19.74±0.29**
**WFH10 (%)**	**98.17±2.27**	**99.96±2.37**	106.27±2.58*	116.87±2.67**	**95.74±2.13**	**97.71±2.02**	**99.39±1.84**	105.86±1.81**	**96.68±1.58**	**98.70±1.57**	102.76±1.57**	110.95±1.58**
Δ **Weight (kg)**	**13.08±0.73**	**13.55±0.76**	**15.15±0.83**	18.39±0.86**	**12.03±0.71**	**12.29±0.67**	**12.87±0.61**	15.80±0.60**	**12.49±0.51**	**12.90±0.51**	13.98±0.51*	17.03±0.51**
Δ **BMI (kg/m** ^**2**^ **)**	**2.13±0.29**	**2.27±0.30**	**2.93±0.33**	3.77±0.34**	**1.84±0.26**	**1.73±0.25**	**1.98±0.23**	2.78±0.22**	**1.95±0.20**	**1.99±0.20**	**2.44±0.20**	3.25±0.20**
Δ **WFH (%)**	**-0.92±1.60**	**0.10±1.67**	**3.69±1.82**	6.07±1.88**	**-2.71±1.56**	**-3.85±1.48**	**-2.59±1.35**	**-0.27±1.33**	**-2.06±1.12**	**-1.91±1.12**	**0.46±1.12**	2.76±1.12**

Abbreviations Weight5: weight at 5 years old; Weight10: weight at 10 years old; ΔWeight: change of weight from 5 to 10 years; BMI5: BMI at 5 years old; BMI10: BMI at 10 years old; ΔBMI: change of BMI from 5 to 10 years; WFH5: WFH at 5 years old; WFH 10: WFH at 10 years old; ΔWFH: change of WFH from 5 to 10 years

*P<0.05

**P<0.05

**Table 3:** Predictors of weight gain in children of early childhood Dependent Variable

Independent variable	β	SE	95%CI	*P* value
**Model A:**				
Gender	-0.2	0.43	(0.35–1.90)	0.65
Birth weight	-0.0004	0	(0.99–1.00)	0.41
TV viewing time	0.41	0.09	(1.26–1.80)	<0.0001
Weight at baseline	0.75	0.06	(1.88–2.38)	<0.0001
Fasting insulin at baseline	0.31	0.07	(1.18–1.56)	<0.0001
**Model B:**				
Gender	-0.54	0.19	(0.40–0.84)	0.005
Birth weight	-0.0001	0.0002	(0.99–1.00)	0.97
TV viewing time	0.17	0.04	(1.10–1.28)	<0.0001
BMI at baseline	0.15	0.05	(1.05–1.28)	0.0052
Fasting insulin at baseline	0.18	0.03	(1.13–1.27)	<0.0001

Model A: Change of weight during 5 years

Model B: Change of weight during 5 years

**Fig 1:**
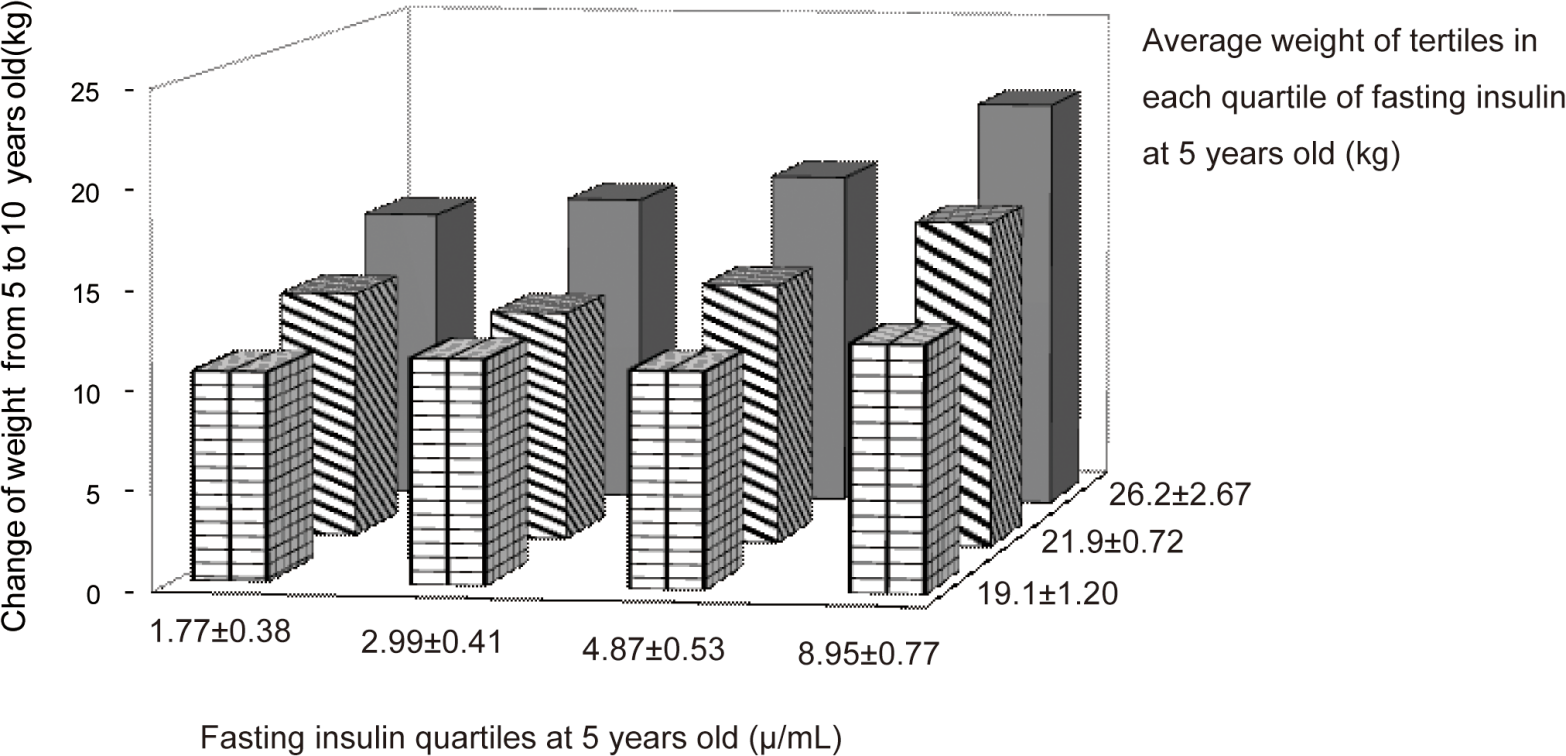
The change of weight during a 5-year follow-up related to the plasma fasting insulin and weight at baseline. With the increase of fasting insulin and weight at baseline, weight increased gradually and became greater. The mean value weight gain during the 5-year follow-up in the top weight at baseline tertile was significant greater than that in the lowest tertile in each fasting insulin at baseline quartile (*p*<0.01).The greatest weight gain was in the top quartile of fasting insulin and meanwhile in the top tertile of weight at baseline. Baseline fasting insulin significantly correlated with ΔWeight in the subgroup of top and middle weight tertile (r = 0.39, *P*<0.0001 and r = 0.33, *P*<0.0001, respectively), but not in the lowest weight tertile subgroups at baseline (r = 14, *P*>0.05).

To further investigate the correlation of ΔWeight over the 5 years with both fasting insulin and weight at baseline, we stratified weight at 5 years into tertiles in each quartile group of fasting insulin at baseline. The results showed that ΔWeight over 5 years significantly increased with increasing weight at baseline in each group of fasting insulin quartiles, and the greatest ΔWeight was found in the group had highest-fasting insulin with highest-weight at baseline (Fig 1). When we compared the differences of cardiovascular risk factors among these subgroups, we found that SBP, FPG, TG and HOMA-IR at 10 years of age were all significantly higher in the highest-fasting insulin with highest-weight group compared to the lowest-insulin with lowest-weight group (SBP: 104.11±1.43 *vs*. 92.91±1.49 mmHg, *p* = 0.0002; FPG: 5.54±0.08 *vs*. 5.19±0.08 mmol/L, *p* = 0.01; TG:1.31±0.07 *vs*. 0.99±0.07 mmol/L, *p* = 0.0005; HOMA-IR:1.70±0.12 *vs*. 0.90±0.13, *p* = 0.0001, data not shown in table).

We also analyzed the relationship between weight at baseline and the change of fasting insulin level during the 5-year follow-up. It was found that the weight at baseline did not predict the change in fasting insulin over 5-year follow-up(r = 0.02, *P*>0.05, did not show in table). The change of fasting insulin in the top weight quartile group was not significantly different with that in the lowest weight group (-0.22±0.40 *vs* 0.54±0.3 (μU/ml); *P*>0.05, data not shown in table).

## Discussion

The present study showed clearly that fasting plasma insulin in early childhood is an independent predictor of subsequent weight gain over a 5-year follow-up period in boys and girls in Da Qing children. This result was also demonstrated by another two indices of weight and obesity—BMI and WFH. By contrast, the weight in early childhood at 5 years old was not significantly correlated with fasting insulin, and was not an important predictor for the increase of fasting insulin levels in subsequent years.

The relationship between insulin concentration and change of weight has been of interest in the last 2 decades. Maffeis et al found that insulin resistance at childhood was related to reduced risk of obesity at adulthood[14]. The findings led to the hypotheses that insulin resistance inhibited glucose storage and glucose oxidation; in addition, some of the authors then implied that it might be correlated with the observation that obese adults might decrease weight gain via behavior and diet control. However, the CARDIA study examined 3095 young adults followed up for 7 years and found that an increase of 5 uU/ml in fasting insulin predicted a 5 kg/m^2^ increase in BMI after adjustment for race and gender [9]. Another relatively large sample size study reported that HOMA-IR significantly predicted total and central adiposity increased after 6 years follow-up in the Swedish children[11]. Johnson et al showed that increased fat mass were significantly associated with fasting insulin concentrations in a cohort of 83 Caucasian and 54 African children[20]. The current study is the first longitudinal study over 5 years in ethnic Chinese children. All the subjects in the study population were in early childhood and were all of the same age, and did not have already had any other metabolic syndrome features (age of enrollment was 5 years). It is reasonable to propose that the present results are a more accurate reflection of the real association between insulin and weight gain. Thus, we suggest that fasting insulin in early childhood is a predictor of weight gain during late childhood (from 5 to 10 years of age), but in adults, hyperinsulinemia is more likely secondary to obesity. Consistent with our present findings, Olalekan’s study of Pima India children followed up for 9.3 years from 5–9 years old to puberty, also found that fasting plasma insulin concentrations at baseline was a predictor of the rate of weight gain independently of the initial relative weight and change in height per year[17], although the Pima Indians children were more obese and their fasting insulin level were higher compared with Da Qing children. The possible potential mechanism of how high insulin levels could enhance weight gain is that hyperinsulinemia may cause a greater energy intake to excessive weight gain in children[21]. Moreover, insulin resistance in the brain co-occur with insulin resistance in the periphery[22].

It is generally considered that obesity is caused by multiple genetic and environmental factors. Previously, Powell’s studies reported that TV viewing time was an important environmental factor on weight gain[23]. In the present study, the multivariate regression analysis had shown that the fasting insulin concentration at baseline still positively correlated with weight gain and the increase of BMI over the 5 years after adjustment of birth weight, TV viewing time and the weight at baseline. This finding further supports our hypothesis that fasting insulin concentration in early childhood may independently affect weight gain.

In addition, obesity is considered a risk factor of cardiovascular risk factors. Some studies in obesity children identified that insulin resistance was a predicator of blood pressure in overweight and obese children[24] and also predicted the development of impaired glucose tolerance and diabetes that occurred at the onset of puberty[10]. In our study, the children with the greatest fasting insulin together with the greatest weight at baseline have significantly greater weight gain and the increased of cardiovascular risk factors: SBP, FPG, TG and HOMA-IR after 5 years follow-up than the children with the lowest fasting insulin combined with the lowest weight at baseline (Fig 1). This suggests that more concern should be given for these children to intervention and prevention at early age.

There were some limitations in the present study. Firstly, only fasting plasma insulin and glucose were examined because candidates were too young and an OGTT was not considered acceptable by the authorities or the parents. Secondly, we are unable to report the relationship between waist circumference and fasting insulin, as we did not test waist circumference because of the big variation of the measurement with breath in children with 5 years of age although we knew that some studies showed that waist or waist-to-height ratio associated with insulin sensitivity[25]. Thirdly, more accurate measurements of body fat were not made as they were not available at the time of study. Fourthly, we did not have a formal assessment of pubertal stage at 10 years of age.

## Conclusions

Fasting plasma insulin in early childhood was a predicator of weight gain from early to late childhood. By contrast, body weight at baseline was not able to predict any change in fasting insulin during a 5-year follow-up. Thus, the children even if at early stage of childhood with relatively higher fasting insulin levels should be given more attention to prevent the development of obesity and the accumulation of risk factors of a potential metabolic syndrome.
